# Reduction of SIRT1 epigenetically upregulates NALP1 expression and contributes to neuropathic pain induced by chemotherapeutic drug bortezomib

**DOI:** 10.1186/s12974-018-1327-x

**Published:** 2018-10-20

**Authors:** Kun Chen, Jing Fan, Zhao-Fan Luo, Ying Yang, Wen-Jun Xin, Cui-Cui Liu

**Affiliations:** 1 The Joint Research Centre of Gene Interference, Guangzhou University and Keele University for Gene Interference and Application, School of Life Science, Guangzhou University, 230 Waihuan West Road, Guangzhou, 510006 China; 2 Department of Clinical Laboratory, The Seventh Affiliated Hospital of Sun Yat-Sen University, Shenzhen, 518017 China; 3 Guangdong Province Key Laboratory of Brain Function and Disease, Department of Physiology and Pain Research Center, Zhongshan School of Medicine, Sun Yet-Sen University, Guangzhou, 510080 China; 4 Guangdong Provincial Key Laboratory of Malignant Tumor Epigenetics and Gene Regulation, Department of Rehabilitation Medicine, Sun Yat-Sen Memorial Hospital, Sun Yat-Sen University, 107 Yanjiang West Road, Guangzhou, 510120 China

**Keywords:** Bortezomib, SIRT1, NALP1, STAT3, Mechanical allodynia

## Abstract

**Background:**

Bortezomib is a frequently used chemotherapeutic drug for the treatment of multiple myeloma and other nonsolid malignancies. Accumulating evidence has demonstrated that bortezomib-induced persistent pain serves as the most frequent reason for treatment discontinuation.

**Methods:**

The von Frey test was performed to evaluate neuropathic pain behavior, and real-time quantitative reverse transcription polymerase chain reaction, chromatin immunoprecipitation, western blot, immunohistochemistry, and small interfering RNA were performed to explore the molecular mechanisms in adult male Sprague-Dawley rats.

**Results:**

We found that application of bortezomib significantly increased the expression of NALP1 protein and mRNA levels in spinal dorsal horn neurons, and intrathecal application of NALP1 siRNA attenuated the bortezomib-induced mechanical allodynia. In addition, bortezomib also decreased the SIRT1 expression, and treatment with SIRT1 activator resveratrol ameliorated the NALP1 upregulation and mechanical allodynia induced by bortezomib. Meanwhile, knockdown of SIRT1 using the SIRT1 siRNA induced the NALP1 upregulation in dorsal horn and mechanical allodynia in normal animal. These results suggested that reduction of SIRT1 induced the NALP1 upregulation in dorsal horn neurons, and participated in bortezomib-induced mechanical allodynia. Importantly, we found that the binding of SIRT1 and NALP1 promoter region did not change before and after bortezomib treatment, but SIRT1 downregulation increased p-STAT3 expression. Furthermore, the activation of STAT3 enhanced the recruitment of p-STAT3 to the *Nalp1* gene promoter, which increased the acetylation of histone H3 and H4 in NALP1 promoter regions and epigenetically upregulated NALP1 expression in the rodents with bortezomib treatment.

**Conclusion:**

These findings suggested a new epigenetic mechanism for NALP1 upregulation involving SIRT1 reduction and subsequent STAT3-mediated histone hyperacetylation in NALP1 promoter region in dorsal horn neurons, which contributed to the bortezomib-induced mechanical allodynia.

## Background

Bortezomib (BTZ), as a first-generation proteasome inhibitor, is primarily used for the treatment of relapsed or resistant multiple myeloma. However, painful neuropathy, one of its dose-limiting toxicities, often results in dose reduction or cessation of the first-line treatment, and it usually persists a long period even after the cessation of treatment [1, 2]. Due to the lack of understanding on the mechanisms underlying bortezomib-induced painful neuropathy, the clinical use of bortezomib is largely limited [2], which necessitates the further research to identify the underlying mechanism.

Evidences showed that inflammasomes are groups of protein complexes that recognize diverse sets of inflammation-inducing stimuli such as pathogenic or tissue damage [3]. NACHT leucine-rich-repeat protein (NALP), as a newly discovered inflammasome family, is composed of the NALP protein, caspase-1, and the adaptor and is implicated in multiple neuroinflammation-related disorders [4]. Notably, studies have demonstrated that the upregulation of peripheral NALP1 inflammasome induces peripheral sensitization and contributes to the complex regional pain syndrome [5, 6]. Furthermore, the increase of NALP1 inflammasome is involved in neuropathic pain induced by chronic constriction injury (CCI) [7]. However, whether the expression of NALP1 inflammasome in dorsal horn contributes to chemotherapeutic drug-induced persisted pain has not been previously described.

Silent information regulator 1 (SIRT1), as a member of sirtuin family, is involved in a wide variety of cellular processes including inflammation, immune, apoptosis, and metabolism [8–10]. In central nerve system, SIRT1 can also modulate synaptic plasticity and memory formation [11, 12]. Recently, SIRT1 has been reported to be involved in the development of chronic pain. Spinal SIRT1 expression was downregulated in the mouse or rat with chronic constriction nerve injury, and SIRT1 activator SRT1720 ameliorated CCI-induced neuropathic pain [13, 14]. However, whether SIRT1 participated in the chemotherapeutic drug-induced persistent pain remains unclear. In addition, previous studies showed that SIRT1 negatively regulated the expression of NLRP3 inflammasome in the traumatic brain injury [15] and vascular endothelial cells [16]. Notably, accumulating evidences have demonstrated that epigenetic modulation of the expression of pain-related inflammatory genes played an important role in the development and maintenance of neuropathic pain [17, 18]. Currently, whether the potential dynamic adaptation of SIRT1 regulates the NALP1 expression in the dorsal horn, and the underlying epigenetic mechanism, remains largely unknown.

## Methods

### Animals

Male Sprague-Dawley rats (200-250 g) and male C57BL/6 mice (20–30 g) were obtained from the Institute of Experimental Animals of Sun Yat-Sen University. *STAT3*^flox/flox^ mice (ID: 016923) were commercially purchased from the Jackson Laboratory. All animals were housed separately under controlled conditions (23 ± 1 °C, 55 ± 3% humidity, 6am to 6pm alternate light/dark cycles) with food and tap water ad libitum. All animals were randomly assigned to different groups. The experimental protocols were approved by Sun Yat-Sen University Animal Care and Use Committee and were carried out in accordance with the National Institutes of Health Guide for the Care and Use of Laboratory Animals. All efforts were made to minimize the number and the suffering of animals used.

### Injection of drug and adenovirus-associated vector

Bortezomib (Haoran Biological Technology CO, Shanghai) was intraperitoneally injected at 0.4 mg/kg once per day for consecutive 5 days as described previously [19]. Control animals received an equivalent volume of vehicle saline. siRNA, which are chemically modified for the enhanced stability with methylation and for high efficiency delivery with cholesterol-conjugation, were commercially obtained from Ribobio. The saline was used to siRNA delivery. Resveratrol (50 μg/10 μl, 250 μg/10 μl, and 500 μg/10 μl; Sigma-Aldrich, USA), NALP1 siRNA (50 μg/10 μl; Ribobio, China), scrambled siRNA (50 μg/10 μl; Ribobio, China), SIRT1 siRNA (50 μg/10 μl; Ribobio, China), S3I-201 (100 μg/10 μl; Selleck Chemicals, USA), or vehicle saline (10 μl) was intrathecally administrated 30 min prior to bortezomib treatment and maintained for 10 days.

4 μl of recombinant adeno-associated virus encoding Cre and green fluorescent protein (GFP) marker (AAV8-Cre-GFP, Beijing Vector Gene Technology Company Ltd.) was intrathecally injected into the subarachnoid space of L5-L6 spinal cord in *STAT3*^flox/flox^ mouse. In the control group, the same amount of AAV8-GFP was intrathecally administrated in *STAT3*^flox/flox^ mouse. Bortezomib was intraperitoneally administrated on day 21 after the injection of virus.

### Lumbar subarachnoid catheterization and behavioral assessment

For intrathecal injection, after animals were anesthetized intraperitoneally with 50 mg/kg sodium pentobarbital, polyethylene intrathecal catheters (PE-10, Becton Dickinson, USA) were implanted into animals as described previously [19]. In brief, the catheter was inserted into the lumbar subarachnoid space between fifth and sixth lumbar vertebrae with the tip of the catheter located near the L5 spinal segmental level. Following intrathecal implantation of catheters, animals were allowed 5 days to recover from surgery prior to subsequent drug injection, and any animals with hind limb paresis or paralysis were excluded from the present study. 10 μl of 2% lidocaine was injected to confirm correct catheter position, as indicated by transient bilateral hind limb paralysis.

Mechanical allodynia was tested using von Frey filaments applied to the plantar region of the hind paw as described previously. Briefly, animals were placed in a plastic box on a metal mesh and were allowed to acclimate for 30 min before testing. von Frey filaments of different bending force were applied alternately to the midplantar surface of hind paw. Mechanical allodynia of each animal was assessed by the hind paw mechanical withdrawal threshold. A nociceptive response was defined as a brisk paw withdrawal or paw flinching following von Frey filament application. Each trial was repeated for three times at 2 min intervals. The 50% paw withdrawal threshold was calculated for each animal following a previous validated up-down procedure [20]. The behavioral tests were conducted by an experimenter who was blinded to all treatments.

### RNA extraction and real-time quantitative PCR

Total RNA was extracted from dorsal horn (L4–L6) tissues by Trizol reagent (Invitrogen, USA). SYBR Green qPCR SuperMix (Invitrogen, USA) and ABI PRISM7500 Sequence Detection System were used for real-time quantitative PCR. The reverse transcription reactions were performed using oligo-dT primer and M-MLV reverse transcriptase (Promega, USA) according to the protocol based on the manufacturer’s instructions. The amount of synthesized cDNA was evaluated by PCR by using primers specific for NALP1 (rat): forward 5′-GTGGCTGGACCTCTGTTTGA-3′, and reverse 5′-GGCGTTTCTAGGACCATCCC-3′. PCR amplification was carried out with an initial denaturing step at 94 °C for 3 min, then 40 cycles at 94 °C for 10 s, at 58 °C for 20 s, and at 72 °C for 10 s. The relative expression ratio of mRNA in L4–L6 dorsal horn tissues was quantified by the 2^−△△CT^ method [21].

### Western blotting

Western blotting was in accordance with the description in our previous study [22]. Briefly, L4–L6 spinal dorsal horn tissues of animals were removed and homogenized in 15 mmol/l Tris containing a cocktail of proteinase inhibitors and phosphatase inhibitors following animals were anesthetized with 50 mg/kg sodium pentobarbital (i.p.). Next, the lysates of L4–L6 dorsal horn tissues were prepared and separated by gel electrophoresis (SDS-PAGE) and transferred onto a polyvinylidene fluoride membrane. They were then pre-incubated for 1 h at room temperature in the block buffer. After incubating with diluted primary antibodies against SIRT1 (1:2000, #ab110304, Abcam, UK), NALP1 (1:1000, #ab3683, Abcam, UK), phosphorylated STAT3 (1:1500, #ab76315, Abcam, UK), acetylated histone H3 (1:1000, #ab8898, Abcam, UK), acetylated histone H4 (1:1000, #07–329, Millipore, USA), or β-actin (1:2000, #ab8226, Abcam, UK) overnight at 4 °C, the membranes were incubated in horseradish peroxidase-conjugated secondary antibody for 1 h at room temperature. Finally, the bands in the membranes were visualized by enhanced chemiluminescence (ECL, Pierce, USA) as directed by the manufacturer. The bands then were quantified with computer-assisted imaging analysis system (NIH ImageJ).

### Immunohistochemistry

Immunohistochemistry was performed as previously described [22]. Briefly, animals were anesthetized with sodium pentobarbital (50 mg/kg, i.p.), and cardiac perfusion were performed using 0.9% physiological saline, followed by 4% paraformaldehyde in PBS. Next, L4–L6 spinal cord tissues were removed and postfixed in the same fixative overnight and then dehydrated with 30% sucrose. Cryostat sections (16 μm thick) were cut and processed for immunofluorescent staining with primary antibodies for SIRT1 (1:400, Santa Cruz, USA), NALP1 (1:50, Novus Biologicals, USA), phosphorylated STAT3 (1:100, Abcam, UK), NeuN (1:500, Chemicon, USA), GFAP (1:800, Chemicon, USA), or OX42 (1:250, Chemicon, USA). After incubation overnight at 4 °C, the sections were then incubated with cy3-conjugated and fluorescein isothiocyanate-conjugated secondary antibodies for 1 h at room temperature. Isotype IgG was applied in separated sections as control, which did not yield detectable immunosignal. The stained sections were then examined with a Leica (Leica, Germany) fluorescence microscope, and images were captured with a Leica DFC350 FX camera.

### Chromatin immunoprecipitation (ChIP) assays

ChIP assays were carried out using the ChIP Assay Kit (Thermo) as described previously [22]. The animals were anesthetized with sodium pentobarbital (50 mg/kg, i.p.), and L4–L6 spinal dorsal horn were removed immediately and placed in 1% formaldehyde for 2 min. Then, DNA was fragmented by sonication and digested with micrococcal nuclease. After adding ChIP dilution buffer into DNA sample, 100 μl of the sample was saved as input. The precleared chromatin solution was incubated with anti-p-STAT3 antibody (Cell Signaling Technology, USA) or anti-acetyl-Histone H3 antibody (Abcam, UK) or anti-acetyl-Histone H4 antibody (Millipore, USA) overnight at 4 °C. The “IgG” immunoprecipitation was applied for the negative control. Next day, DNA was purified from the immunecomplexes and input fractions following the antibody/DNA complexes were captured, washed, eluted, and reverse cross-linked. The purified DNA was then resuspended in the nuclease-free water, and real-time quantitative PCR was performed on the sample as described in the above methods. Finally, the ratio of ChIP/input in the L4–L6 spinal dorsal horn was calculated. Primers 5′-CTCAATTCATGGTATTCTAAG-3′ and 5′-GAAGAAATTCATACATCACTG-3′ were designed to amplify a − 1148/− 1057 region relative to the transcription start site of rat *Nalp1* promoter, containing the STAT3-binding site. Primers 5 -TCAAACACCCATTCACAGAGC-3 and 5 -CAAGACGCTCTTCTGTTGTTG-3 were designed to amplify a − 139/− 55 region relative to the transcription start site of mouse *Nalp1* promoter, containing the STAT3-binding site.

### Coimmunoprecipitation

Coimmunoprecipitation was performed using the Co-Immunoprecipitation Kit (Thermo Fisher Scientific, USA) as described previously [22]. Briefly, animals were anesthetized with intraperitoneal injection of sodium pentobarbital (50 mg/kg), and L4–L6 spinal dorsal horn tissues were excised and homogenized in lysis buffer. The anti-p300 antibody (Abcam, UK) or anti-p-STAT3 antibody (Abcam, UK), which immobilized with resin, were used to collect the immune complexes. The eluted complexes from the resin were then analyzed by western blotting using anti-p-STAT3 antibody or anti-p300 antibody after incubation and washes.

### Statistical analysis

All data were expressed as the mean values ±SEM, and analyzed with SPSS 20.0 (SPSS, USA). For behavioral tests, one-way or two-way ANOVA with repeated-measures followed by a Tukey post hoc test was carried out. Western blotting and real-time quantitative PCR data were analyzed by two-way ANOVA followed by a Tukey post hoc test. The criterion for statistical significance was *P* < 0.05. While no power analysis was performed, the sample size was determined according to our and peers’ previous publications in painful behavior and pertinent molecular studies.

## Results

### Upregulation of NALP1 contributed to bortezomib-induced mechanical allodynia

**Fig. 1:**
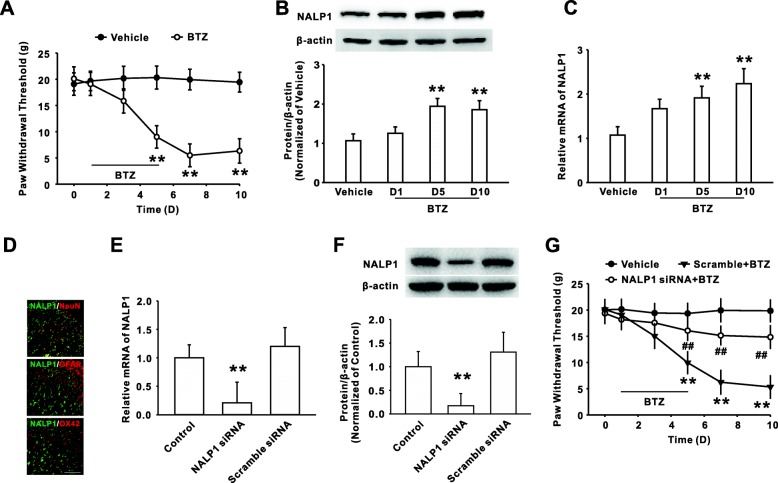
Increased NALP1 in spinal dorsal horn is involved in bortezomib-induced mechanical allodynia. **a** Intraperitoneal administration of bortezomib (0.4 mg/kg for five consecutive days) markedly decreased the mechanical withdrawal threshold. *n* = 12 in each group, **P < 0.01 versus vehicle group. The level of NALP1 protein (**b**) and mRNA (**c**) in dorsal horn was significantly upregulated following BTZ treatment. *n* = 6 in each group, **P < 0.01 versus the vehicle group. **d** The photographs showed the double staining between neuronal markers (NeuN, red) or astrocyte marker (GFAP, red) or microglia marker (OX42, red) with NALP1 (green) in spinal cord of rats on day 10 following BTZ treatment. *n* = 4 in each group; scale bar, 50 μm. The expression of NALP1 mRNA (**e**) and protein (**f**) in dorsal horn were suppressed on day 10 following consecutive intrathecal injection of NALP1 siRNA for 10 days. *n* = 5 in each group, **P < 0.01 versus corresponding scramble siRNA group. **g** Intrathecal delivery of NALP1 siRNA for 10 days attenuated the mechanical allodynia following BTZ treatment. *n* = 12 in each group, **P < 0.01 versus vehicle group, ^##^*P* < 0.01 versus corresponding scramble siRNA group

Consistent with our previous study [23], intraperitoneal administration of bortezomib (0.4 mg/kg for five consecutive days) markedly decreased the mechanical withdrawal threshold (Fig. 1a). The results of western blot and PCR showed that the expression of dorsal horn NALP1 protein and mRNA was significantly enhanced on day 5 and 10 following bortezomib treatment (Fig. 1b, c). Double immunofluorescent staining showed that the increased NALP1 was primarily colocalized with NeuN (a neuronal marker), but not with GFAP (an astrocytic marker) or Iba-1 (a microglial marker) (Fig. 1d). To define the role of NALP1 in bortezomib-induced persistent pain, NALP1 siRNA was continuously administrated into the naive rats for 10 days (50 μg/10 μl, i.t.), which significantly decreased the levels of NALP1 mRNA (Fig. 1e) and protein (Fig. 1f) on day 10. Further behavioral study found that knockdown of NALP1 in dorsal horn by intrathecal injection of the NALP1 siRNA significantly attenuated the mechanical allodynia induced by bortezomib (Fig. 1g).

### Reduction of SIRT1 upregulated NALP1 expression and participated in bortezomib-induced mechanical allodynia

**Fig. 2:**
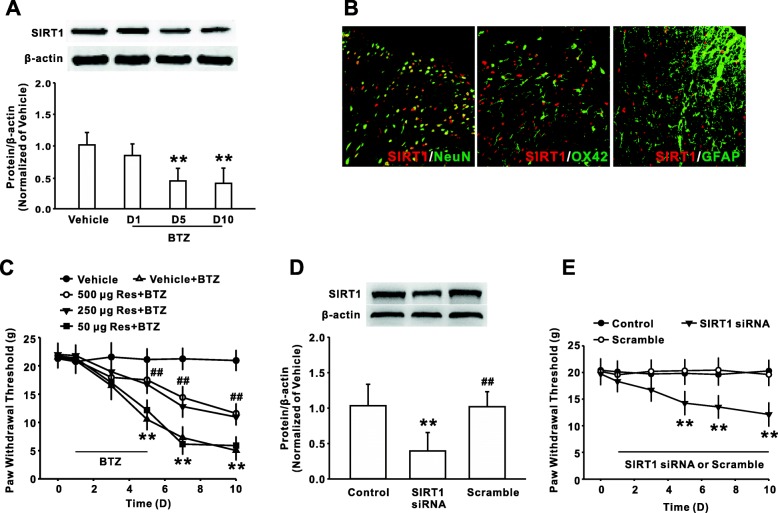
The decrease of SIRT1 in spinal dorsal horn participated to bortezomib-induced mechanical allodynia. **a** Representative histogram and blots revealed the decrease of SIRT1 in spinal dorsal horn following BTZ treatment. *n* = 6 in each group, **P < 0.01 versus the vehicle group. **b** The photographs showed double staining between neuronal markers (NeuN, green) or astrocyte marker (GFAP, green) or microglia marker (OX42, green) with SIRT1 (red) in spinal cord of rats on day 10 following BTZ treatment. *n* = 3 in each group; scale bar, 100 μm. **c** Continuous intrathecal injection of resveratrol at dose of 500 μg/10 μl or 250 μg/10 μl, but not 50 μg/10 μl for 10 days ameliorated mechanical allodynia following BTZ treatment. *n* = 12 in each group, **P < 0.01 versus the vehicle group, ^##^*P <* 0.01 versus corresponding vehicle + bortezomib group. **d** Intrathecal injection of SIRT1 siRNA downregulated the SIRT1 expression in dorsal horn. *n* = 3 in each group, **P < 0.01 the control group, ^##^*P* < 0.01 versus corresponding siRNA group. **e** Downregulation of the SIRT1 by SIRT1 siRNA induced the mechanical allodynia in normal rats. *n* = 8 in each group, **P < 0.01 the control group

Next, we found that the dorsal horn SIRT1 protein expression was significantly decreased on days 5 and 10 following bortezomib treatment (Fig. 2a). Double immunofluorescent staining showed that SIRT1 was primarily expressed in NeuN-positive cells, but not GFAP-positive and OX42-positive cells (Fig. 2b). To further confirm the role of SIRT1 in bortezomib-induced persistent pain, we investigated the behavioral response to the artificial manipulation of SIRT1 function in dorsal horn. The results showed that intrathecal injection of specific SIRT1 activator resveratrol at dose of 250 μg or 500 μg, but not 50 μg, for consecutive 10 days attenuated the mechanical allodynia induced by bortezomib (Fig. 2c). Meanwhile, we specifically downregulated the expression of spinal SIRT1 in normal rats by intrathecal administration of SIRT1 siRNA for consecutive 10 days and then assessed pain behavior. The knockdown efficiency of SIRT1 siRNA was validated by western blotting (Fig. 2d). Compared with the control group, intrathecal injection of siRNA (50 μg/10 μl) induced mechanical allodynia in the naïve rats (Fig. 2e).

**Fig. 3:**
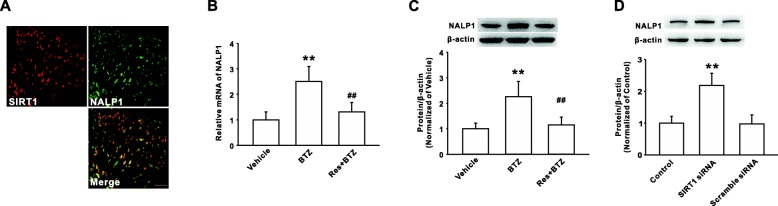
SIRT1 modulated the NALP1 expression following bortezomib treatment. **a** The photographs show double staining between SIRT1 (red) with NALP1 (green) in spinal dorsal horn on day 10 following bortezomib treatment. *n* = 4 in each group; scale bar, 50 μm. Intrathecal injection of resveratrol (250 μg/10 μl for 10 days) inhibited the upregulation of NALP1 mRNA (**b**) and protein (**c**) level in dorsal horn of rats on day 10 following BTZ treatment. *n* = 6 in each group, **P < 0.01 versus the vehicle group, ^##^*P* < 0.01 versus the corresponding BTZ group. **d** Intrathecal injection of SIRT siRNA increased the NALP1 expression in normal rats. *n* = 6 in each group, **P < 0.01 versus the control group

We next determined whether SIRT1 modulated the NALP1 expression in dorsal horn in the rodents with bortezomib treatment. Firstly, the double immunostaining results showed that SIRT1 was co-expressed with NALP1 in spinal dorsal horn in the rats with bortezomib treatment (Fig. 3a). Furthermore, SIRT1 activation with resveratrol (250 μg/ 10 μl, i.t. for 10 days) prevented the upregulation of NALP1 mRNA and protein on day 10 following bortezomib treatment (Fig. 3b, c). Meanwhile, compared with the control rats, the expressions of NALP1 were upregulated in the rats with intrathecal injection of SIRT1 siRNA (Fig. 3d). These findings suggested that reduction of SIRT1 induced the increases of NALP1 expression and subsequently contributed to bortezomib-induced mechanical allodynia.

### STAT3 activation induced by SIRT1 reduction contributed to the expression of NALP1

**Fig. 4:**
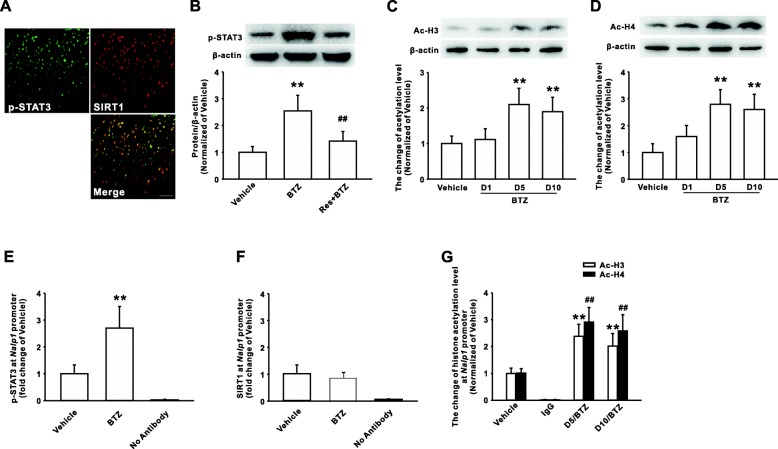
P-STAT3, but not SIRT1, directly mediated the expression of NALP1 following bortezomib treatment. **a** The photographs show double staining between SIRT1 (red) with NALP1 (green) in spinal dorsal horn on day 10 following bortezomib treatment. *n* = 4 in each group; scale bar, 50 μm. **b** Application of resveratrol (i.t.) reduced the increase of p-STAT3 on day 10 following BTZ treatment. *n* = 6 in each group, **P < 0.01 versus the vehicle group, ^##^*P* < 0.01 versus the corresponding BTZ group. BTZ treatment significantly increased the global acetylation of histone H3 (K9) (**c**) and H4 (K16) (**d**) in spinal dorsal horn of rats. *n* = 6 in each group, **P < 0.01 versus vehicle group. **e** ChIP results showed the enhanced recruitment of p-STAT3 to Nalp1 gene promoter on day 10 following BTZ treatment. *n* = 5 in each group, **P < 0.01 versus vehicle group. **f** The recruitment of SIRT1 to Nalp1 gene promoter did not significantly changed on day 10 in spinal dorsal horn of rats with bortezomib treatment. *n* = 5 in each group, **P < 0.01 versus vehicle group. **g** ChIP assay showed BTZ treatment increased the acetylation of histone H3 and H4 on Nalp1 gene promoter region flanking p-STAT3-binding site in rats. *n* = 6 in each group, **P < 0.01 versus corresponding vehicle group, ^##^*P* < 0.01 versus the corresponding vehicle group

Studies showed the activated STAT3 may increase the histone acetylation in promoter region and enhance the expression of inflammation-associated genes [22, 24], and our previous study indicated that activation of STAT3 contributed to bortezomib-induced mechanical allodynia [23]. In the present study, we found that p-STAT3 was expressed in SIRT1-positive cells in spinal dorsal horn (Fig. 4a), and SIRT1 activator resveratrol significantly inhibited the increase of spinal p-STAT3 induced by bortezomib (Fig. 4b). Further immunoblotting results showed that global acetylation level of H3 (k9) and H4 (k16) was increased in dorsal horn following bortezomib treatment (Fig. 4c, d). Previous studies reported that SIRT1, as a kind of nicotinamide adenine dinucleotide (NAD1)-dependent deacetylase, can preferentially regulate the acetylation of H3 (k9) and H4 (k16) [25, 26]. Then, we determined whether the histone hyperacetylation resulted from the reduced SIRT1 deacetylase activity or the increased p-STAT activity induced by SIRT1 reduction in dorsal horn in the rats with bortezomib treatment. The TFSEARCH and the JASPAR database’ analysis showed that the position − 1127/− 1117 region of *Nalp1* gene exists a potent-binding site for STAT3. Then, DNA precipitated by p-STAT3 antibody or SIRT1 antibody was proceeded to PCR analysis, in which the primers was designed to amplify a 92-bp fragment (− 1148/− 1057) of *Nalp1* promoter flanking STAT3-binding site. The results revealed that the recruitment of p-STAT3 (Fig. 4e), but not SIRT1 (Fig. 4f), to *Nalp1* promoter region was significantly enhanced on day 10 in spinal dorsal horn of rats with bortezomib treatment, compared with the vehicle-treated rats. Importantly, the ChIP assay also revealed that H3 (k9) and H4 (k16) acetylation level were increased on the *Nalp1* promoter region flanking the p-STAT3-binding site after treatment with bortezomib on day 10 (Fig. 4g). These findings suggested that NALP1 upregulation results from STAT3-mediated histone acetylation on the *Nalp1* promoter region flanking the p-STAT3-binding site rather than from a direct action of the SIRT1 reduction after bortezomib treatment.

### Interaction between pSTAT- and P300-mediated histone hyperacetylation in *Nalp1* promoter region induced by bortezomib

**Fig. 5:**
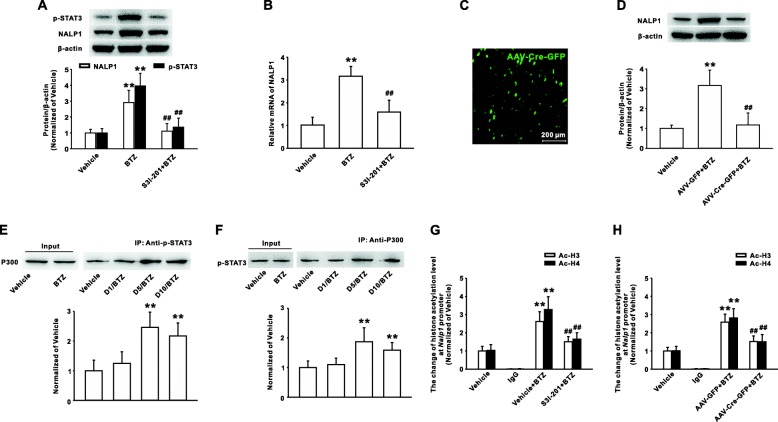
Activated STAT3 by binding p300 increased the level of acetylated histone H3and H4 on the Nalp1 promoter. Continuous injection of STAT3 activity inhibitor S3I-201 (100 μg/10 μl for 10 days, i.t.) inhibited the upregulation of NALP1 mRNA (**a**) and protein (**b**) level on day 10 following BTZ treatment. *n* = 8 in each group, **P < 0.01 versus the vehicle group, ^##^*P* < 0.01 versus the corresponding BTZ group. **c** A marked green fluoresces in the dorsal horn of mice was observed on day 21 after AAV8-Cre-GFP injection. **d** Local deficiency of STAT3 by intrathecal injection of AAV-Cre-GFP in STAT3^flox/flox^ mice significantly reduced the upregulation of NALP1 protein in spinal dorsal horn induced by BTZ treatment. *n* = 6 in each group, **P < 0.01 versus vehicle group, ^##^*P* < 0.01 versus the corresponding AAV-GFP group. **e** Increased p300 was significantly immunoprecipitated with p-STAT3 antibody in spinal dorsal horn of rats following BTZ administration. *n* = 6 in each group, **P < 0.01 versus vehicle group. **f** Increased p-STAT3 was significantly immunoprecipitated with P300 antibody on the different time points after bortezomib treatment. *n* = 6 in each group, **P < 0.01 versus vehicle group. **g** Intrathecal application of S3I-201 reduced the increase of acetylated H3 or H4 on Nalp1 gene promoter region containing p-STAT3-binding site on day 10 following BTZ treatment. *n* = 6 in each group, **P < 0.01 versus vehicle group, ^##^*P* < 0.01 versus the corresponding BTZ group. **h** Local deficiency of STAT3 by intrathecal injection of AAV-Cre-GFP into STAT3^flox/flox^ mice decreased the upregulation of H3 or H4 acetylation on Nalp1 gene promoter induced by BTZ. *n* = 6 in each group, **P < 0.01 versus vehicle group, ^##^*P* < 0.01 versus the corresponding AAV-GFP injected BTZ group

Next, we further explored the mechanism of STAT3-regulating NALP1 expression. Consecutive intrathecal injections of STAT3 inhibitor S3I-201, while decreasing the phosphorylation of STAT3 (Fig. 5a), significantly reduced the NALP1 upregulation at protein (Fig. 5a) and mRNA (Fig. 5b) levels in dorsal horn induced by bortezomib.

A marked green fluoresces in the dorsal horn of mice on day 21 after AAV8-Cre-GFP injection suggested a high efficiency of transfection (Fig. 5c). Conditional knockout of STAT3 by intrathecal administration of AAV-Cre-GFP in *STAT3*^flox/flox^ mice also inhibited the upregulation of NALP1 protein level induced by bortezomib (Fig. 5d). Furthermore, the co-immunoprecipitation (Co-IP) results showed that bortezomib treatment markedly increased the histoneacetyl transferase p300 content in the immunocomplex precipitated by p-STAT3 antibody (Fig. 5e). Similarly, the increased p-STAT3 content at the different time points was also observed in the immunocomplex precipitated by p300 antibody in the dorsal horn lysates. These results confirmed that bortezomib enhanced the interaction between p-STAT3 and p300 (Fig. 5f). Next, The ChIP assay revealed that intrathecal injection of S3I-201 into rats or AAV-Cre-GFP into *STAT3*^flox/flox^ mice significantly inhibited the bortezomib-induced H3 and H4 hyperacetylation on *Nalp1* promoter region flanking the p-STAT3-binding site (Fig. 5g, h). Taken together, these results suggested that bortezomib induced an enhancement of histone acetylation at the promoter of *Nalp1* gene through increasing interaction of p-STAT3 and p300.

## Discussion

In the present study, we first found that reduction of SIRT1 induced activation of STAT3, and subsequently upregulated the expression of NALP1 in dorsal horn, and contributed to the mechanical allodynia induced by chemotherapeutic drug bortezomib. Bortezomib increased the NALP1 expression in spinal dorsal horn neurons, and intrathecal application of NALP1 siRNA attenuated the bortezomib-induced mechanical allodynia. In addition, bortezomib treatment decreased the SIRT1 expression in dorsal horn neuron, and intrathecal injection of SIRT1 activator resveratrol ameliorated the NALP1 upregulation and mechanical allodynia following bortezomib treatment. Moreover, knockdown of SIRT1 using the SIRT1 siRNA increased the NALP1 expression and induced mechanical allodynia in normal animal. These results demonstrated the critical role of SIRT1 reduction in NALP1 upregulation in dorsal horn and pain behavior induced by bortezomib. We further found that SIRT1 reduction increased the phosphorylation of the transcript factor STAT3 in dorsal horn in the rodents with bortezomib treatment. We also found that interaction between p-STAT and p300 in *Nalp1* promoter region enhanced the histone acetylation and facilitated the expression of NALP1 in dorsal horn in the rodents with bortezomib treatment. Taken together, these findings suggested a new mechanism by which SIRT1 regulated NALP1 expression in dorsal horn neurons and subsequently contributed to bortezomib-induced mechanical allodynia.

In our study, we observed for the first time that application of chemotherapeutic drug bortezomib upregulated NALP1 protein and mRNA expression in dorsal horn, and intrathecal injection of NALP1 siRNA attenuated the mechanical allodynia induced by bortezomib. It is well known that NALP1 inflammasome is responsible for the activation of pro-inflammatory caspases [4], and inhibition of NALP1 inflammasome activation significantly decreases IL-1β maturation and Pro-IL-1β synthesis [27]. So, it is possible that inflammatory cytokines such as IL-1β induced by NALP1 inflammasome significantly contribute to the painful neuropathy induced by bortezomib. It was consistent with the previous evidence that spinal NALP1 inflammasome regulated spinal IL-1β maturation and contributed to the development of CCI-induced neuropathic pain [28]. While the possibility existed that the analgesia induced by intrathecal dosing scheme in the present study might be partially supplemented with its potential effect on DRG neurons or their central terminals, our results at least showed that the spinal cord NALP1 signaling was critically involved in the BTZ-induced mechanical allodynia.

The present study also showed that SIRT1 was also involved in the painful neuropathy induced by chemotherapeutic drug bortezomib, as evidenced by our findings that recovery of SIRT1 activity with resveratrol attenuated the bortezomib-induced mechanical allodynia. Moreover, artificial suppression of SIRT1 activity by specific siRNA significantly decreased the withdrawal threshold in naive rats. Similar findings were reported by the previous studies that the SIRT1 expression and activity were decreased in the spinal cord in rodent models with CCI, and modulations of SIRT1 activity by resveratrol ameliorated the development of CCI-induced neuropathic pain [13, 14, 29]. Study also showed that the SIRT1 downregulation increased H3 acetylation levels at Grm1/5 promoter region, consequently enhanced the mGluR1/5 expression, and contributed to the neuropathic pain in type 2 diabetic rats. Notably, although the present study showed that the SIRT1 downregulation promoted the NALP1 expression, bortezomib did not decrease the SIRT1 binding at NALP1 promoter regions flanking the p-STAT3-binding site relative to the control group, suggested that the increases of NALP1 might not directly result from the reduced deacetylase activity following SIRT1 decrease induced by bortezomib. Our previous and peer’s studies showed that STAT3, a transcriptional factor, was involved in the neuropathic pain induced by nerve injury or chemotherapeutic drug [19, 23, 30]. Furthermore, studies showed that SIRT1 suppressed STAT3 phosphorylation, which promoted the transcription of gluconeogenic gene [31]. In the present study, we found that the activation of SIRT1 by resveratrol significantly inhibited STAT3 phosphorylation and decreased the recruitment of p-STAT3 to the *Nalp1* promoter, which suppressed the expression of NALP1 in the spinal dorsal horn of rats following bortezomib treatment. In conclusion, SIRT1 reduction induced STAT3-mediated epigenetic upregulation of NALP1 in dorsal horn and contributed to the persistent pain induced by chemotherapeutic drug bortezomib.

## Conclusions

Taken together, our data demonstrated a new epigenetic mechanism by which SIRT1 regulated NALP1 expression in dorsal horn neurons and subsequently contributed to bortezomib-induced mechanical allodynia. Importantly, the NALP1 upregulation resulted from STAT3-mediated histone acetylation on the *Nalp1* promoter region that was induced by SIRT1 reduction after bortezomib treatment.

## Abbreviations

BTZ: Bortezomib
CCI: Chronic constriction injury
IL-1β: Interleukin-1β
mRNA: Messenger RNA
NALP1: NACHT leucine-rich-repeat protein
siRNA: Small interference RNA
SIRT1: Silent information regulator 1
STAT3: Signal transducers and transcription activator 3

## References

[1] Ale A, Bruna J, Navarro X, Udina E (2014). Neurotoxicity induced by antineoplastic proteasome inhibitors. Neurotoxicology.

[2] Miltenburg NC, Boogerd W (2014). Chemotherapy-induced neuropathy: a comprehensive survey. Cancer Treat Rev.

[3] Strowig T, Henao-Mejia J, Elinav E, Flavell R (2012). Inflammasomes in health and disease. Nature.

[4] Tschopp J, Martinon F, Burns K (2003). NALPs: a novel protein family involved in inflammation. Nat Rev Mol Cell Biol.

[5] Shi X, Wang L, Li X, Sahbaie P, Kingery WS, Clark JD (2011). Neuropeptides contribute to peripheral nociceptive sensitization by regulating interleukin-1beta production in keratinocytes. Anesth Analg.

[6] Li WW, Guo TZ, Liang D, Shi X, Wei T, Kingery WS, Clark JD (2009). The NALP1 inflammasome controls cytokine production and nociception in a rat fracture model of complex regional pain syndrome. Pain.

[7] Li Q, Tian Y, Wang ZF, Liu SB, Mi WL, Ma HJ, Wu GC, Wang J, Yu J, Wang YQ (2013). Involvement of the spinal NALP1 inflammasome in neuropathic pain and aspirin-triggered-15-epi-lipoxin A4 induced analgesia. Neuroscience.

[8] Li Y, Wang P, Yang X, Wang W, Zhang J, He Y, Zhang W, Jing T, Wang B, Lin R (2016). SIRT1 inhibits inflammatory response partly through regulation of NLRP3 inflammasome in vascular endothelial cells. Mol Immunol.

[9] Kauppinen A, Suuronen T, Ojala J, Kaarniranta K, Salminen A (2013). Antagonistic crosstalk between NF-kappaB and SIRT1 in the regulation of inflammation and metabolic disorders. Cell Signal.

[10] Michan S, Sinclair D (2007). Sirtuins in mammals: insights into their biological function. Biochem J.

[11] Gao J, Wang WY, Mao YW, Graff J, Guan JS, Pan L, Mak G, Kim D, Su SC, Tsai LH (2010). A novel pathway regulates memory and plasticity via SIRT1 and miR-134. Nature.

[12] Michan S, Li Y, Chou MMH, Parrella E, Ge HY, Long JM, Allard JS, Lewis K, Miller M, Xu W (2010). SIRT1 is essential for Normal cognitive function and synaptic plasticity. J Neurosci.

[13] Lv C, Hu HY, Zhao L, Zheng H, Luo XZ, Zhang J (2015). Intrathecal SRT1720, a SIRT1 agonist, exerts anti-hyperalgesic and anti-inflammatory effects on chronic constriction injury-induced neuropathic pain in rats. Int J Clin Exp Med.

[14] Shao H, Xue Q, Zhang F, Luo Y, Zhu H, Zhang X, Zhang H, Ding W, Yu B (2014). Spinal SIRT1 activation attenuates neuropathic pain in mice. PLoS One.

[15] Zou P, Liu X, Li G, Wang Y (2018). Resveratrol pretreatment attenuates traumatic brain injury in rats by suppressing NLRP3 inflammasome activation via SIRT1. Mol Med Rep.

[16] Li Y, Yang X, He Y, Wang W, Zhang J, Zhang W, Jing T, Wang B, Lin R (2017). Negative regulation of NLRP3 inflammasome by SIRT1 in vascular endothelial cells. Immunobiology.

[17] Denk F, Huang W, Sidders B, Bithell A, Crow M, Grist J, Sharma S, Ziemek D, Rice AS, Buckley NJ, McMahon SB (2013). HDAC inhibitors attenuate the development of hypersensitivity in models of neuropathic pain. Pain.

[18] Pan Z, Shan Q, Gu P, Wang XM, Tai LW, Sun M, Luo X, Sun L, Cheung CW (2018). miRNA-23a/CXCR4 regulates neuropathic pain via directly targeting TXNIP/NLRP3 inflammasome axis. J Neuroinflammation.

[19] Liu CC, Huang ZX, Li X, Shen KF, Liu M, Ouyang HD, Zhang SB, Ruan YT, Zhang XL, Wu SL (2018). Upregulation of NLRP3 via STAT3-dependent histone acetylation contributes to painful neuropathy induced by bortezomib. Exp Neurol.

[20] Chaplan SR, Bach FW, Pogrel JW, Chung JM, Yaksh TL (1994). Quantitative assessment of tactile allodynia in the rat paw. J Neurosci Methods.

[21] Rao X, Huang X, Zhou Z, Lin X (2013). An improvement of the 2^(−delta delta CT) method for quantitative real-time polymerase chain reaction data analysis. Biostat Bioinforma Biomath.

[22] Xu T, Zhang XL, Ou-Yang HD, Li ZY, Liu CC, Huang ZZ, Xu J, Wei JY, Nie BL, Ma C (2017). Epigenetic upregulation of CXCL12 expression mediates antitubulin chemotherapeutics-induced neuropathic pain. Pain.

[23] Wei JY, Liu CC, Ouyang HD, Ma C, Xie MX, Liu M, Lei WL, Ding HH, Wu SL, Xin WJ (2017). Activation of RAGE/STAT3 pathway by methylglyoxal contributes to spinal central sensitization and persistent pain induced by bortezomib. Exp Neurol.

[24] Zhong Z, Wen Z, Darnell JE (1994). Stat3: a STAT family member activated by tyrosine phosphorylation in response to epidermal growth factor and interleukin-6. Science.

[25] Imai S, Armstrong CM, Kaeberlein M, Guarente L (2000). Transcriptional silencing and longevity protein Sir2 is an NAD-dependent histone deacetylase. Nature.

[26] Feige JN, Auwerx J (2008). Transcriptional targets of sirtuins in the coordination of mammalian physiology. Curr Opin Cell Biol.

[27] Liu S, Li Q, Zhang MT, Mao-Ying QL, Hu LY, Wu GC, Mi WL, Wang YQ (2016). Curcumin ameliorates neuropathic pain by down-regulating spinal IL-1beta via suppressing astroglial NALP1 inflammasome and JAK2-STAT3 signalling. Sci Rep.

[28] Stephan AH, Madison DV, Mateos JM, Fraser DA, Lovelett EA, Coutellier L, Kim L, Tsai HH, Huang EJ, Rowitch DH (2013). A dramatic increase of C1q protein in the CNS during normal aging. J Neurosci.

[29] Yin Q, Lu FF, Zhao Y, Cheng MY, Fan Q, Cui J, Liu L, Cheng W, Yan CD (2013). Resveratrol facilitates pain attenuation in a rat model of neuropathic pain through the activation of spinal Sirt1. Reg Anesth Pain Med.

[30] Lu J, Zhang L, Chen X, Lu Q, Yang Y, Liu J, Ma X (2014). SIRT1 counteracted the activation of STAT3 and NF-kappaB to repress the gastric cancer growth. Int J Clin Exp Med.

[31] Nie Y, Erion DM, Yuan Z, Dietrich M, Shulman GI, Horvath TL, Gao Q (2009). STAT3 inhibition of gluconeogenesis is downregulated by SirT1. Nat Cell Biol.

